# A novel crosslinking agent of polymethyl(ketoxime)siloxane for room temperature vulcanized silicone rubbers: synthesis, properties and thermal stability

**DOI:** 10.1039/c7ra13375h

**Published:** 2018-04-03

**Authors:** Xibing Zhan, Xiqing Cai, Junying Zhang

**Affiliations:** College of Chemical and Material Engineering, Quzhou University Zhejiang 324000 China; Lab of Adhesives and In-situ Polymerization Technology, Key Laboratory of Carbon Fiber and Functional Polymers, Ministry of Education, Beijing University of Chemical Technology Beijing 100029 China zjybuct@gmail.com +86 10 64425439 +86 10 64425439

## Abstract

A novel cross-linker polymethyl(ketoxime)siloxane (PMKS) with dense pendant reactive groups based on polymethylhydrosiloxane (PMHS) was synthesized *via* dehydrocoupling reaction. The novel PMKS cross-linker was applied to a hydroxyl-terminated polydimethylsiloxane (HPDMS) matrix to prepare a series of novel RTV silicone rubbers. The chemical structure of PMKS and curing reaction between HPDMS and PMKS by hydrolytic condensation were verified by IR spectroscopy and ^1^H NMR. Thermal stability and mechanical properties of these novel RTV silicone rubbers have been studied by means of thermal gravimetric analysis (TGA) and universal tensile testing machine, respectively. The results displayed that a pronounced enhancement effect of the novel cross-linker PMKS on thermal stabilities and mechanical properties of RTV silicone rubbers as compared with the traditional cross-linking agent of methyltris(methylethylketoximino)silane (MTKS). Subsequently, the degradation residues were also characterized by FT-IR and X-ray photoelectron spectrometer (XPS). It was found that the striking enhancements in thermal properties and improvements on mechanical properties could be the synergistic effect of the T-type branched structure of PMKS cross-linker, *in situ* formation of dense PMKS phase in the chain network by self-crosslinking and the uniform distribution of PMKS cross-linker in the HPDMS matrix.

## Abstract 2

A novel cross-linker polymethyl(ketoxime)siloxane was synthesized and then was cured with hydroxyl-terminated polydimethylsiloxane matrix to fabricate a series of novel RTV silicone rubber. Their properties was comparatively investigated.

## Graphical abstract

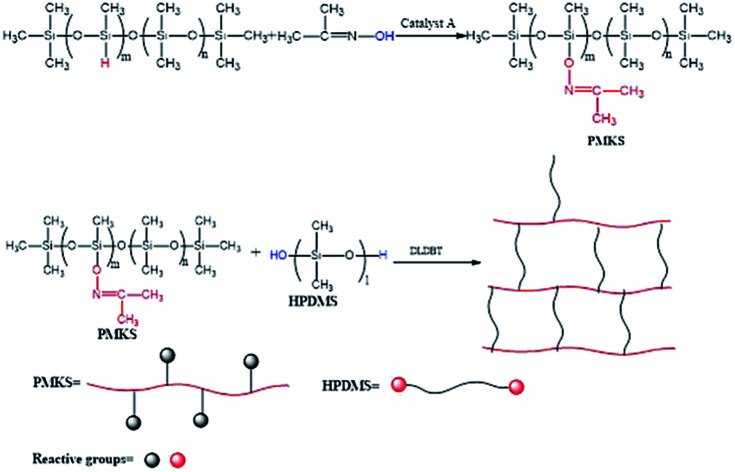

## 1. Introduction

Silicone materials, which are semi-inorganic polymers, are widely used as a key matrix resin in many applications from biomaterials to coating and sealants, *et al.*^1–7^ They have some unique physical and chemical properties such as remarkable thermal and thermo oxidative resistance, weather and solvent resistance, biological inertness and much better resistance to electromagnetic and particle radiation (UV, alpha, beta and gamma rays) than organic plastics.^8–10^ The silicone materials can be availed mainly three routes: tin- or titanium-catalyzed room temperature vulcanization (moisture cure), platinum-catalyzed hydrosilylation addition cure and radical cure which is normally performed at higher temperature.^11–13^

As the most common member of the polysiloxane family, polydimethylsiloxane (PDMS) has been shown to be thermally unstable above 300 °C under vacuum.^14^ At elevated temperatures, the polysiloxane materials will undergo a dramatic chemical change such as the rearrangement of molecular bonds.^15^ So far, a variety of techniques and processes have been reported on the improvement in thermal resistance of polysiloxane materials at elevated temperatures. One of the most popular techniques is the composition or hybridization with some heat-resistant groups and elements, including phenyl,^16,17^ fluorene and adamantine,^18,19^ polysilsesquioxane (POSS),^20–26^ and boron elements in siloxane chains,^27,28^ or inorganic additive blending (such as silica, Al_2_O_3_ and montmorillonite clay, *etc.*) with polysiloxane compounds.^29–32^ The incorporation of methyl-phenyl siloxane or diphenyl siloxane as a copolymer with PDMS has been shown to increase the onset temperature of degradation to nearly 400 °C.^16^ Most of the methods mentioned above focused on the incorporation of heat-resistant and rigid groups or inorganic additives. Nevertheless, these polysiloxanes with heat-resistant organic groups generated a few complex degradation products which were difficult to identify and analyze, and the degradation mechanisms were very various and complicated at high temperature.^33,34^ The inorganic additives are not compatible or hard to disperse in polysiloxane polymer. Some investigation showed that the use of concentrative crosslinking might be one of the effective methods to improve the mechanical strength of siloxane elastomer.^35^ However, very little attention had been paid to improvement of thermal resistance by enhancement of crosslinking network structure. In the past few years, our research team has been carried out some studies on polysiloxane crosslinking agents with many pendant alkoxy groups (PMOS) instead of traditional small crosslinking reagents (such as tetraethoxysilane).^11,36^ This new cross-linkers can be cured with atmospheric moisture and form 3D networks that had much more thermal resistance (6% mass loss at 600 °C for product of PMOS self-crosslinking) compared to the polysiloxane incorporated with phenyl groups. But the tack free time of this system including PMOS crosslinker and hydroxyl-terminated polydimethylsiloxane (HPDMS) by dealcoholization was much longer than traditional curing systems of deacidification and deketoximization. Herein, it is necessary to explore a new macromolecular crosslinking agent with plenty of pendant active groups combined with good thermal stability and high curing reactivity on the basis of PMOS compounds.

The present work is mainly concerned with thermal stability and mechanical properties of novel room temperature vulcanized (RTV) silicone materials. Thus, a new kind of cross-linking agent polymethyl(dimethylketoxime)siloxane (PMKS) with dense pendant ketoxime groups was synthesized by means of dehydrocoupling reaction, and cured with HPDMS compounds in the moisture environment *via* hydrolysis–condensation to form three-dimensional cross-linking networks. We also investigated the mechanical properties and thermal stabilities (including thermal degradation process and degradation residues by TGA, infrared spectroscopy and XPS) of the novel silicone rubber. It is found that PMKS cross-linker was favorable to enhancement in the thermal stabilities and mechanical properties of the novel RTV silicone rubbers in comparison with traditional crosslinking agent methyltris(methylethylketoximino)silane (MTKS).

## 2. Experimental

### 2.1 Materials

Hydroxyl-terminated polydimethylsiloxane (HPDMS, *M*_n_ = 3.6 × 10^4^ g mol^−1^) and polymethylhydrosiloxane (PMHS, *M*_n_ = 4200 g mol^−1^) were supplied by Dow Corning Corporation (USA). Methyltris(methylethylketoximino)silane (MTKS) and dibutyltin dilaurate (DLDBT) were provided from TCI (Shanghai) Chemical Industry Development Co., Ltd. (China). Acetoxime and tetrahydrofuran (THF) were purchased from Sinopharm Chemical Reagent Co., Ltd (China), and dried over calcium hydride (CaH_2_) for 12 h and then purified by distillation. Tetramethylammonium siloxanolate (HO–[Si(CH_3_)_2_–O]_*n*_–N(CH_3_)_4_, catalyst A) was prepared in our laboratory according to the literature.^37^

### 2.2 Synthesis of polymethyl(ketoxime)siloxane (PMKS)

**Scheme 1:**
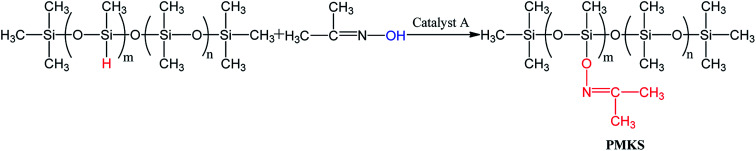
Synthetic route of polymethyl(ketoxime)siloxane (PMKS).

The light yellow and transparent polymethyl(ketoxime)siloxane(PMKS) liquid was synthesized by the dehydrocoupling reaction of PMHS and dimethylketoxime with an amount of catalyst in THF solvent (Scheme 1). The synthetic procedure was as follows according to previous patent:^38^ a mixture of acetoxime (35 g) and THF (200 mL) were charged into a three-necked flask with condenser, thermometer and nitrogen inlet, and stirred until the acetoxime was completely solved. Subsequently, polymethylhydrosiloxane (25 g) and catalyst A (0.48 g) were added to the bottle and stirred at 30 °C for 14 h. When the reaction was completed, all volatiles were removed under vacuum and target products were obtained.

### 2.3 Preparation of room temperature vulcanized (RTV) silicone rubber

**Scheme 2:**
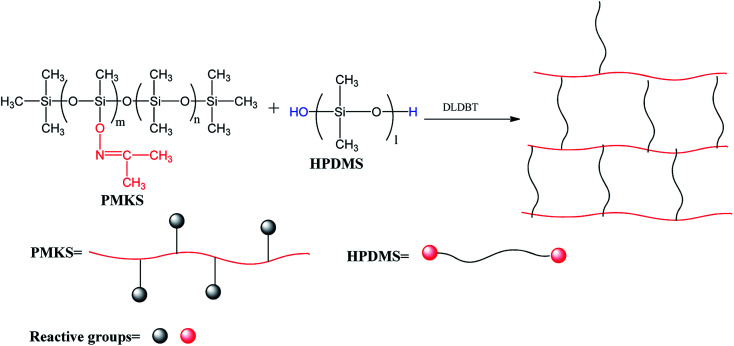
Crosslinking reaction of PMKS with HPDMS.

A series of novel RTV silicone rubbers with various weight portions of PMKS cross-linker were prepared through hydrolysis-condensation between Si–ON=C(CH_3_)_2_ groups of PMKS with Si–OH groups of HPDMS (Scheme 2) under moisture in the presence of catalyst. The process is very sensitive to changes in several factors, such as the concentration of curing catalyst, weight ratio of cross-linker to HPDMS, moisture and temperature. Hence, the weight ratio of catalyst to HPDMS and the preparation conditions were kept constant, only changing the loading levels of PMKS in the formula. The compositions of RTV silicone rubbers were listed in Table 1. The general preparation procedure is as follows:

**Table 1:** Compositions of samples from S1, S3, S4, S5 and S10

Sample	PDMS/g	PMKS/g	MTKS^a^/g	Cross-linker (wt%)
S1	100	5	0	4.76
S3	100	25	0	20
S4	100	35	0	25.9
S5	100	45	0	31
S10	100	0	33.03	24.8

a The mol% of reactive sites of MTKS (methylethylketoximino) (S10) was equal to the mol% of acetoxime groups in PMKS (S5).

**Fig. 1:**
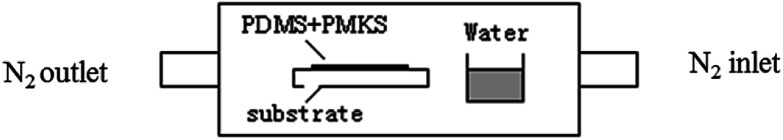
Scheme of the curing equipment for HPDMS/PMKS.

The catalyst of 0.3 wt% dibutyltin dilaurate was added to the mixture of PMKS (or MTKS) and hydroxyl-terminated PDMS and stirred by mechanical agitator, and then was cured in the following curing equipment (Fig. 1) for 36 h at 25 °C. The curing equipment is a glass container in which the mixture of PMKS (or MTKS)/HPDMS/catalyst is poured into a Teflon mould, a bottle of water about 10 mm depth is added to keep at 100% relative humidity and nitrogen was purged at intervals through inlet and outlet to remove the small molecule from the condensation reaction between HPDMS and PMKS (MTKS).

The samples S10 as reference materials were designed to compare the difference between traditional cross-linker such as methyltris(methylethylketoximino)silane (MTKS) and PMKS, in which the mole content of methylethylketoximino is the same as that of acetone oxime in sample of S5.

### 2.4 Characterization

Fourier Transform Infrared (FT-IR) spectra were recorded by Nicolet spectrometer (Nexus 670, USA) ranging from 3500 to 500 cm^−1^ with a resolution of 4 cm^−1^ using KBr pellets. ^1^H NMR measurements were carried out with a nuclear magnetic resonance spectrometer (Bruker AV400, Germany) at 25 °C using CDCl_3_ as solvent.

Elemental composition of the degradation residues were got by X-ray photoelectron spectrometer (XPS) (ESCA Lab 250, UK) using a standard Al Kα source in the analysis chamber (operating conditions: 15 kV voltage, 20 mA current and 2 × 10^7^ Pa pressure) during the experiments.

Thermogravimetric curves (TGA) were obtained using a Thermogravimeter (NETZSCH STA 449C, Germany) ranging from 50 °C to 800 °C with a heating rate of 10 °C min^−1^ in N_2_.

Five specimens with dumb-bell shape were fabricated according to GB/T 528-2009 standard. Tensile tests were conducted using a universal testing machine (CMT4104, China) at speed of 10 mm min^−1^. The Young modulus was determined from the initial slope of the stress–strain curve (1–5% strain range of stress–strain curve).

## 3. Results and discussion

### 3.1 Synthesis and characterization of PMKS and novel RTV silicone rubbers

The synthesis of novel cross-linking agent PMKS is an important stretch to prepare RTV silicone rubbers. In this work, we have successfully synthesized PMKS through dehydrocoupling reaction between Si–H bonds of PMHS and the compounds containing E–H bonds (E = O, N, S, –C=N, *etc.*) by the elimination of hydrogen molecules. To this date, most of researchers are mainly focused on the dehydrocoupling between alcohol and hydrosilane (or hydrosiloxane),^39,40^ and the reaction between other active hydrogen (like NH_2_, HS and C=N–OH) and Si–H is seldom investigated. Hence, we prepare a novel crosslinker PMKS by dehydrogenation between acetoxime and PMHS to vulcanize silicone rubber at 25 °C.

**Fig. 2:**
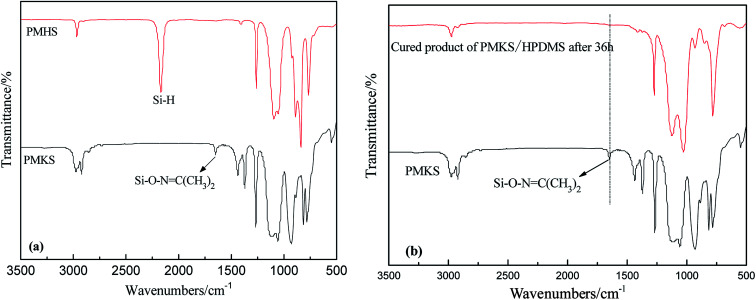
FT-IR spectra of PMHS and PMKS (a); FT-IR spectrum of curing reaction of HPDMS/PMKS (b).

**Fig. 3:**
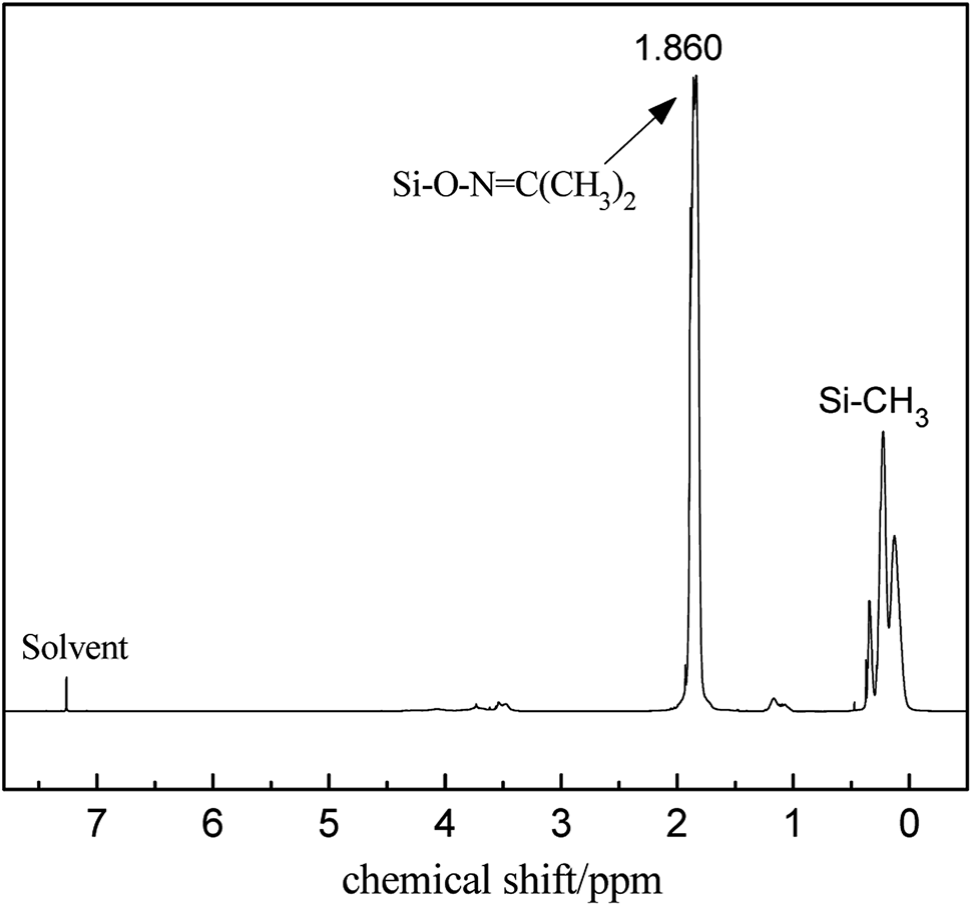
^1^H NMR spectrum of PMKS.

The FT-IR spectra of PMHS and PMKS are shown in Fig. 2(a). From spectrum of PMHS to spectrum PMKS, the signal of Si–H group (2250 cm^−1^) disappears while that of Si–O–N=C(CH_3_)_2_ group (1650 cm^−1^) emerges. These observations seem to reflect the occurrence of dehydrocoupling reaction between PMHS and acetoxime. As we all known, the chemical shift of the Si–H proton of PMHS was at around 4.6 ppm. In Fig. 3, this signal disappeared and a new signal at 1.86 ppm appeared which belonged to the proton of Si–ON=C(CH_3_)_2_ units of PMKS. Therefore, these data from both FT-IR and ^1^HNMR spectra can indicate that the dehydrogenation between PMHS and acetoximes occurs and the molecular structure of PMKS is the same as expected.

The crosslinking reaction of RTV silicone rubber can be monitored by attenuated total reflection infrared (ATR-IR) spectroscopy on the silicone elastomeric surface. The FT-IR spectra of cured PDMS/HPDMS system were dramatically changed in comparison with that of PMKS, and the difference were easily discerned in Fig. 2(b). It is reported that a broad peak at 3448 cm^−1^ and a weak peak at 1630 cm^−1^ of HPDMS spectrum are attributed to stretching and deformation vibration of silanol (Si–OH), respectively.^20^ Two peaks mentioned above completely diminished in the spectrum of cured product. Additionally, the signal at 1650 cm^−1^ belonging to Si–O–N=C(CH_3_)_2_ group of PMKS utterly disappeared after HPDMS was cured and the liquid mixture of PMKS/PDMS becomes elastic solid, which suggested that the silanol groups had reacted completely with cross-linker and new cross-linking network of Si–O–Si had formed in the matrix of PDMS. Moreover, the tack free time (*t*_t_) which referred to the surface cure time, namely the surface tackiness disappeared when touching the surface with fingers, at a certain temperature and humidity according to GB/T 13477.5-2002, and full cure time (*t*_f_) was 120 min, 36 h for HPDMS/PMKS system, 300 min, 48 h for HPDMS/PMOS system and 110 min, 34 h for HPDMS/MTKS in the presence of same amount of catalyst and reactive groups of cross-linking agent, respectively. It implies that the PMKS has much higher reactivity than PMOS, and is similar reactivity to MTKS.

### 3.2 Thermal resistance and pyrolysis reaction of PMKS/HPDMS

PMKS can be used as a novel cross-linker for hydroxyl-terminated (HPDMS) with moisture at 25 °C. To evaluate the *in situ* improvement of this novel cross-linking reagent for HPDMS/PMKS, the thermal degradation and thermal oxidative properties of RTV silicone rubbers as a function of PMKS content were investigated by thermal gravimetric analysis experiments.

**Fig. 4:**
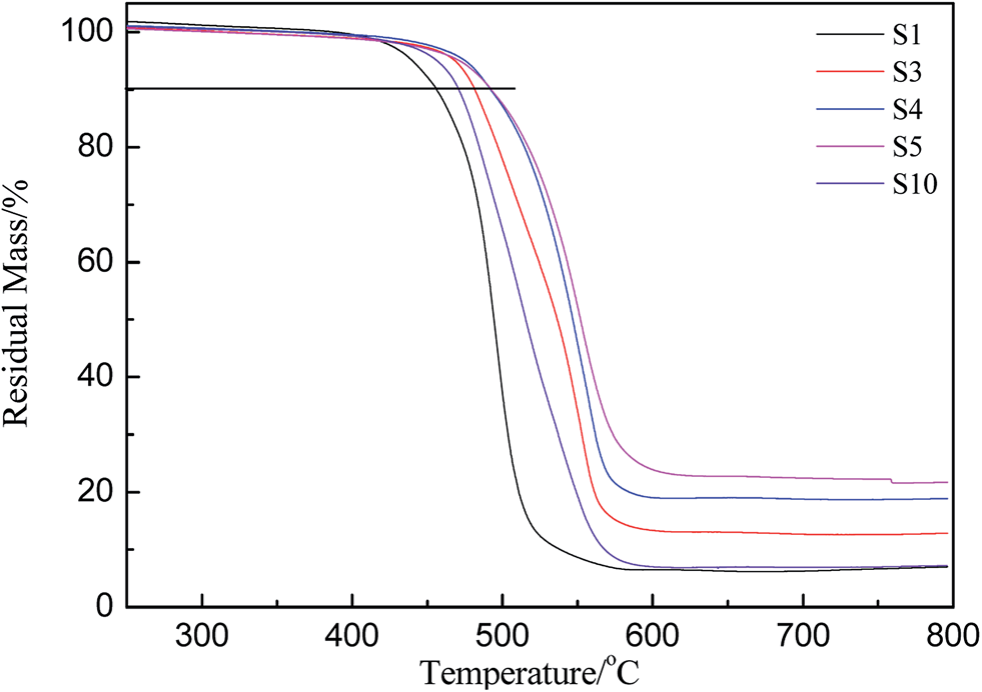
Thermogravimetric curves of crosslinked siloxanes with different weight percent of PMKS and MTKS.

The thermogravimetric curves of HPDMS/PMKS system with different amounts of PMKS (from S1 to S5) and MTKS/HPDMS in N_2_ were shown in Fig. 4 and the residual masses at different temperature were summarized in Table 2. The characteristic temperature of 10% weight loss for novel RTV silicone rubber was ranged from 455.6 °C (S1) to 492.4 °C (S5), which was much higher than that of HPDMS/MTKS (470.7 °C for S10) except for S1 because the content of PMKS crosslinker in S1 was far less than that of S10. The residual mass at 400 °C showed little difference, but the residual mass at 500 °C was very different and the value was 64.8%, 77.2%, 86.8%, 87.3% and 35.2% corresponding to S1, S3, S4, S5 and S10, respectively. The TG curves exhibited that the residual mass for HPDMS/PMKS system rose at 500 °C with an increase in the content of PMKS. Similarly, the residual mass at 600 °C and 800 °C exhibited the same trends, and all of PMKS/HPDMS (except for S1) silicone rubbers were of higher decomposition temperature and more residual mass than MTKS/HPDMS (S10) system. From the above discussion, it is clearly found that PMKS cross-linker has a significant enhancement on thermal stability of the PDMS polymer system relative to conventional cross-linkers.

**Table 2:** Residual mass at different temperature of PMKS/HPMDS system

Sample	Residual mass at different temperature (%)			
	400 °C	500 °C	600 °C	800 °C
S1	99.6	35.2	6.5	6
S3	98.9	77.2	13.3	12.9
S4	99.5	86.8	19.1	18.9
S5	98.8	87.3	23.9	21.8
S10	99.2	64.8	7.3	7.2

**Fig. 5:**
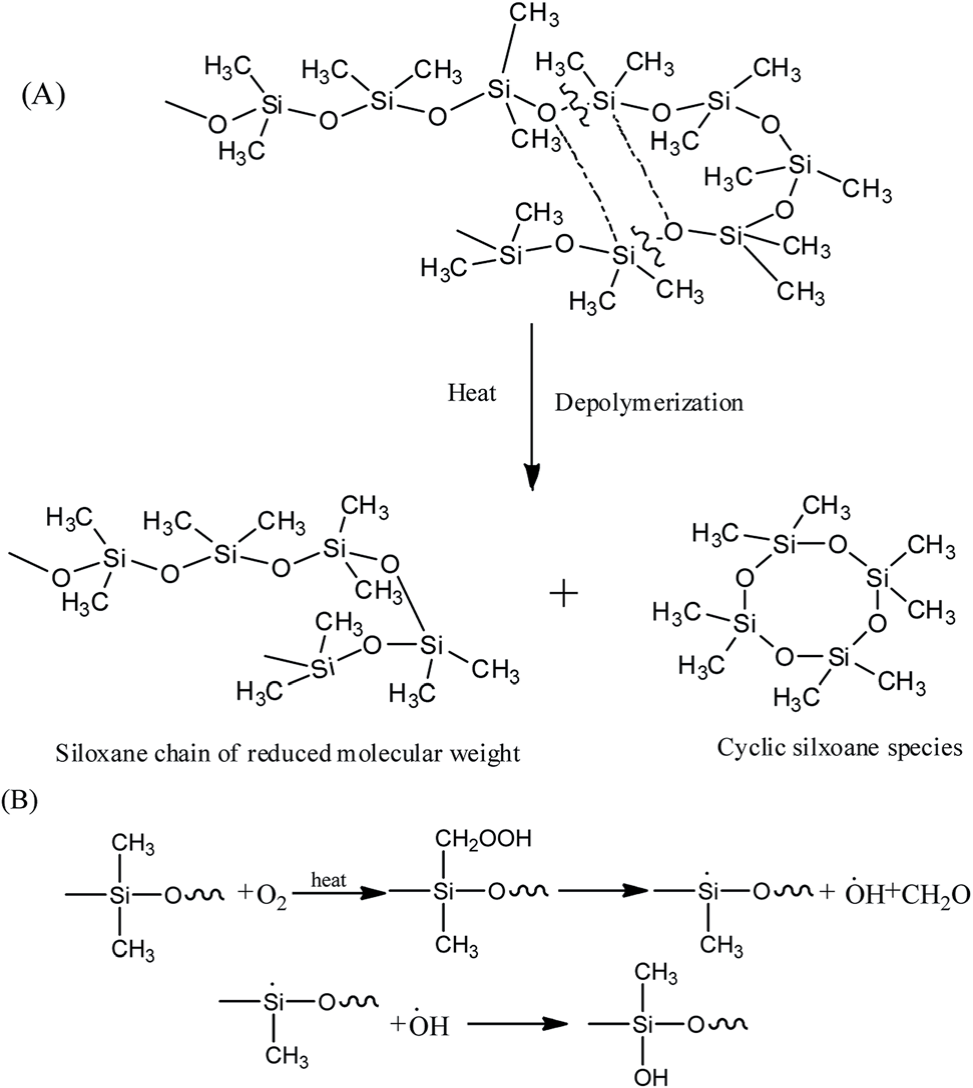
Degradation of main chain (A) and oxidation of the methyl groups of polysiloxane (B).

It's well known that polysiloxane undergoes stepwise degradation of the backbone and oxidation of the methyl groups above 300 °C (Fig. 5).^41,42^ Although the Si–C bond is thermodynamically less than the Si–O bond, thermal degradation of polysiloxane occurs by depolymerization through the Si–O bonds rearrangement, leading to the production of cyclic oligomers.^14,43^ PMKS is a branched molecule with rich pendant acetoxime groups, which maybe has the ability to destroy the helical coiling structure of polysiloxane and form T structure units to make the network compact.^11^ The dense network can constrain the motion of Si–O–Si chain segment. Therefore, the branched and T-type structure of PMKS can prevent the rearrangement of Si–O bonds in polysiloxane and the cyclic oligomers can be blocked. Moreover, the Si–CH_3_ bond scission at higher temperature will form some radical and these macro-radicals may also cross-link by coupling each other.^42^ Steric hindrance and the cross-linked networks of the three-dimensional macro-radicals decrease the flexibility of the PDMS chain, prevent splitting of cyclic oligomers still further and retard further degradation of the PDMS chain.^44^ As a result, PMKS/HPMDS (S3, S4 and S5) have better thermal resistance than MTKS/HPDMS (S10) and the thermal resistance increases with the PMKS contents.

**Fig. 6:**
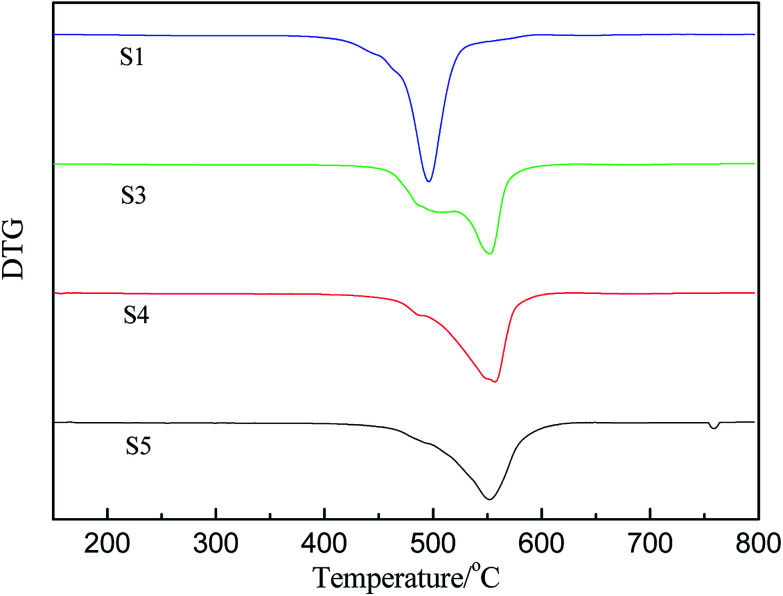
The derivative curves of TGA of crosslinked HPDMS with different contents of PMKS.

The derivative curves of TGA of PMKS/HPMDS (S1, S3, S4 and S5) are presented in Fig. 6. It can be found that there are two degradation peaks, including a shoulder and a maximum degradation peak, for PMKS/HPMDS from Fig. 6, which indicated the existence of different degradation mechanisms. With increment of PMKS content, the intensity of maximum peaks at 500 °C gradually weakened (S1) to be a shoulder (S3, S4 and S5), and the original shoulder peak at 550 °C gradually strengthened (S1) to be a maximum peak (S3, S4 and S5). Thus, the first step degradation maybe corresponds to the decomposition of structures like PDMS networks, while the second step degradation may be related to the PMKS content.^11^ The reaction order and decomposition activation energy can be calculated to evaluate the rate of the new degradation (the second step) behavior of HPDMS with different PMKS content.

The pyrolysis reaction order (*n*) can be calculated by Kissinger method for the non-isothermal degradation:1
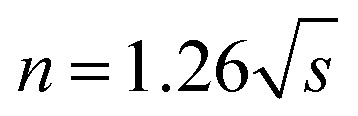
where *s* is the absolute value of the slope at the inflection point (*T*_M_) in the DTG curve.

A dimensionless parameter (*α*) can be defined as follows:2
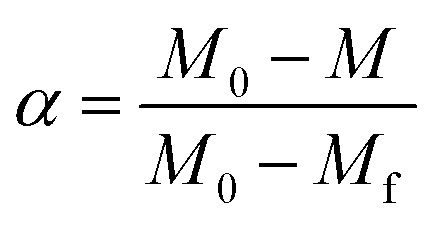


In eqn (2), *M*, *M*_0_ and *M*_f_ represent the sample weight at different temperature, the initial sample weight at 25 °C and the steady-state weight at 800 °C, respectively.

Additionally, a typically kinetic equation can be expressed as:3
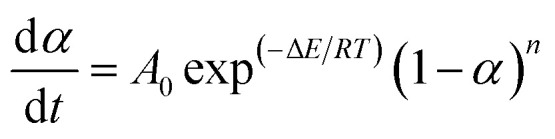
where Δ*E* and *T* stand for degradation temperature and degradation activation energy, respectively. While *A*_0_, *R* is the frequency factor and gas constant, respectively.

Eqn (4) is an integral form of eqn (3) with the initial condition of *α* = 0 at *T* = *T*_0_ and can be expressed as follows4
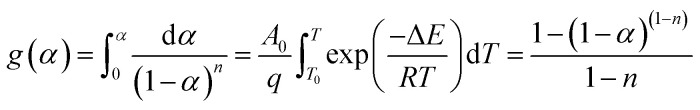
where *q* is the heating rate (d*T*/d*t*).^45^

For the temperature (*T*) ranging from 0.9 *T*_M_ to 1.1 *T*_M_ (*T*_M_ obtained from the peak of DTG curve), the following approximation is made by Van Krevelen method5
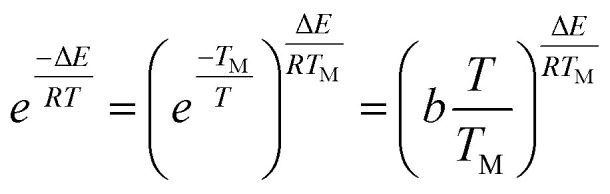
where *b* is a constant equal to 0.368.^46^ Thus, eqn (4) can be integrated for *n* ≠ 1 to6
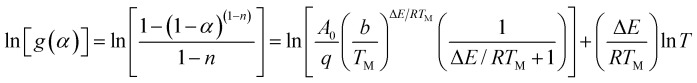


A slope can be obtained from the plot of ln[*g*(*α*)] against ln *T*, and then the activation energy can be calculated.

Reaction parameters for the second degradation of S3, S4 and S5 are calculated and summarized in Table 3 according to Kissinger's method eqn (1) and Van Krevelen eqn (6). The decomposition activation energy increase from 98.3 kJ mol^−1^ to 135.5 kJ mol^−1^ with the PMKS contents increasing at a heating rate of 10 °C min^−1^ maybe because the mobility of the molecular chain is restrained. Camino^47^ have made a research about the correlation between activation energy and heating rate for polysiloxane and found that the activation energy was inversely proportional to heating rate and ranged from 54 up to 250 kJ mol^−1^ in the degradation process. The activation energy measured between 98 and 135 kJ mol^−1^ for PMKS cross-linked HPDMS may be reasonable compared with those values mentioned above.

**Table 3:** Reaction parameter for the second degradation step of PMKS/HPDMS

Sample	Shape index, *s*	*T*_M_/(°C)	Reaction order, *n*	Δ*E* (kJ mol^−1^)
S3	0.107	559.5	0.462	98.3
S4	0.106	563.4	0.432	121.7
S5	0.099	571.3	0.459	135.5

### 3.3 FT-IR and XPS analysis for thermal degradation of PMKS/HPDMS

**Fig. 7:**
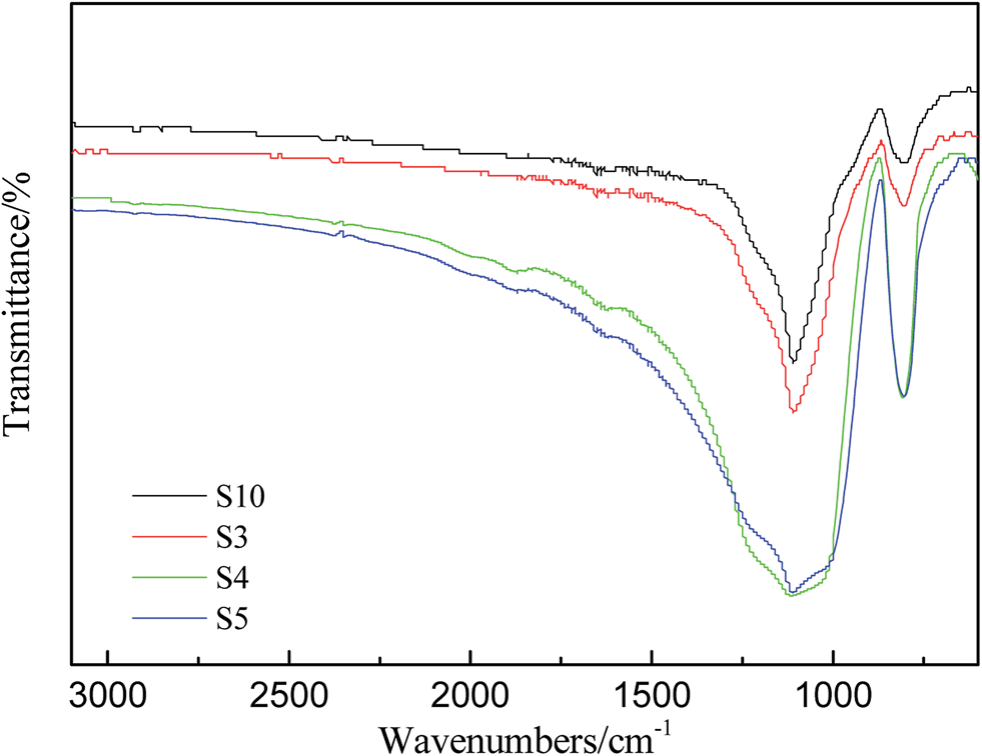
IR spectra of degradation residues of S3, S4, S5 and S10.

It's well known that the thermo-oxidative behavior of the silicone rubber was obviously more complex than the aforementioned thermal degradation behavior in nitrogen atmosphere. FT-IR analysis was conducted to investigate the changes of chemical functionality from the residues of RTV silicone rubber that were kept at 800 °C for two hours in air atmosphere. FT-IR spectra of degradation residues of S3, S4, S5 and S10 are displayed in Fig. 7.

All of the IR spectra of solid degradation residues exhibited a similar trend, and the strongest bands in the spectra are assigned to the asymmetric Si–O–Si vibration between 1086 and 1012 cm^−1^. Although the characteristic stretching vibration peak of C–H of Si–CH_3_ in the vicinity of 2964 cm^−1^ in Fig. 2(b) is indiscernible in Fig. 7, the characteristic bending vibration peak of Si–C at 798 cm^−1^ is discernible. It indicates that the degradation residues in air contain the same compositions. The black degradation residues in N_2_ are mainly siliconoxycarbide,^44,48^ but the degradation residues in air are the grayish white powder which consists of mainly white silica and small amount of black siliconoxycarbide.^48^ Furthermore, the Si–O–Si peaks remain but change from double peaks to one broad single peak at 1120 cm^−1^, which indicates that the Si–O long polymer chains have cleaved (Fig. 7). The bands at 788 cm^−1^ assigned to Si–CH_3_ in S4 and S5, however, are much higher than that in spectra of S10. It may be concluded that PMKS can reduce the oxidation degradation of carbon element in polydimethylsiloxane. The observed improvement effect of different contents of PMKS on thermal stability of silicone rubber is ascribed to the T-type branched structure of PMKS. In other words, PMKS has more than 16 active sites in the side chain as compared with the traditional tri-functional cross-linkers (MTKS), which facilitates formation of 3D networks during the curing process. The three-dimensional network of PDMS polymers decrease the flexibility of the PDMS chain, retard motion of polymer chain and block the formation of cyclic oligomers, and hence elevate the decomposition temperature of the PDMS polymer.

**Fig. 8:**
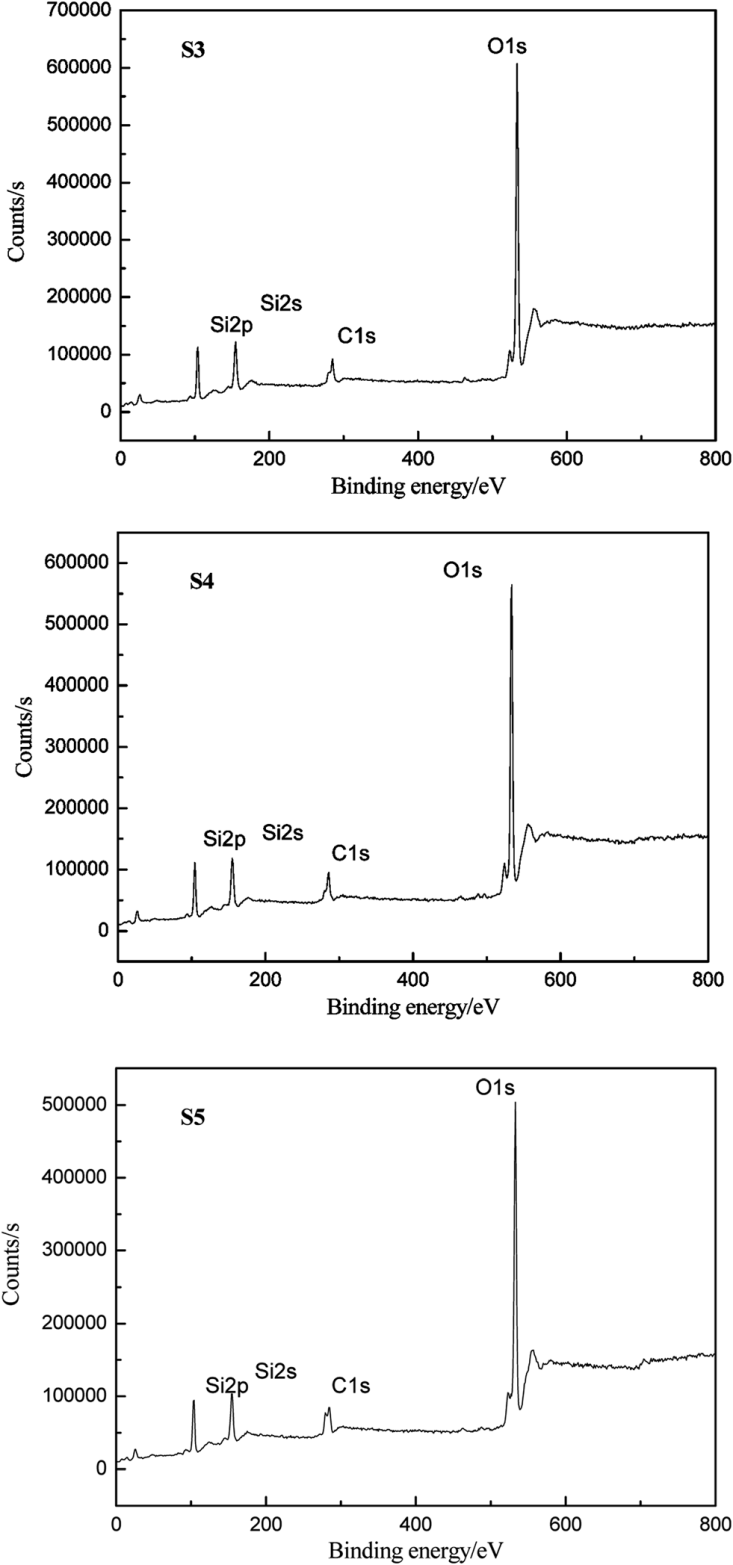
XPS spectra of S3, S4 and S5 for C1s, O1s and Si2p elements after degradation.

The elemental compositions of HPDMS/PMKS bulk containing different PMKS contents (S3, S4 and S5) after 800 °C degradation were recorded by XPS which can detect the chemical element with the order of 1–5nm, to verify the conjecture that PMKS could depress the oxidation degradation of carbon portion in polysiloxane. The XPS spectra of S3, S4 and S5 for C1s, O1s and Si2p regions were illustrated in Fig. 8, and each of the atomic ratio (C/O, C/Si and Si/O) from XPS analysis was summerized in Table 4. In general, the polydimethylsiloxane materials could undergo a dramatic chemical change after 800 °C degradation in air and carbon element could disappear mainly leaving SiO_2_. However, the carbon element didn't totally disappear but still remained in degradation residues, and the C/Si ratio value for S3, S4 and S5 was 0.72, 0.74 and 0.82, respectively. The value of C/Si ratio gradually increased with the PMKS contents rising which indicated that the PMKS could reduce the oxidation degradation of carbon portion. This result was fairly consistent with data from IR analysis.

**Table 4:** Elemental composition (mol%) and atomic ratio from XPS analysis after degradation

Sample	C	O	Si	Si/O ratio	C/Si ratio	C/O ratio
S3	18.92	55.01	26.08	0.47	0.72	0.34
S4	20.82	51.18	28.00	0.54	0.74	0.41
S5	23.52	48.12	28.48	0.59	0.82	0.49

### 3.4 Mechanical properties and average crosslink density of PMKS/HPDMS

To investigate mechanical reinforcement effect of HPDMS polymer system incorporating with PMKS, the mechanical properties of RTV silicone rubbers with different weight fraction of PMKS cross-linker were assessed and the results of mechanical properties such as tensile strength and elongation at break of samples are presented in Table 5. The tensile strength and elongation at break improves greatly as the RTV silicone rubber prepared with PMKS cross-linker (S5), compared with traditional tri-functional cross-linker (MTKS) (S10). It may be because that the PMKS has many reactive groups at the molecular chain relative to MTKS should allow for improved three-dimensional network formation in these PDMS polymers. In addition, it is obviously found that the tensile strength and elongation at break reach a maximum of 0.86 MPa, 276% at first, and then they begin to drop from 0.86 MPa to 0.46 MPa and from 276% to 116% as the content of PMKS exceeded 20 wt% (S3), respectively. S3 sample has the best mechanical properties among all of these samples, which likely result from plasticization of self-crosslinking of PMKS cross-linker in addition to the forming of special three-dimensional networks.^49,50^ In contrast, a large amount of PMKS relative to terminal hydroxyl groups of HPDMS unlikely favors the uniform distribution of PMKS cross-linker during the course of chain growth of the HPDMS units through condensation with the PMKS so that it make an adverse effect on mechanical properties of these polymers, such as S4 and S5 sample. Based on the analysis mentioned above, it is concluded that PMKS can improve the mechanical properties of the HPDMS system.

**Table 5:** Mechanical parameters of S1, S3, S4, S5 and S10

Sample	Tensile strength (MPa)	Elongation at break (%)	Density (g cm^−3^)	Modulus (MPa)	Crosslink density (10^−3^ mol kg^−1^)
S1	0.38	222	0.89	0.28	0.79
S3	0.86	276	0.95	0.34	1.35
S4	0.47	134	1.01	0.51	1.45
S5	0.46	116	1.01	0.56	1.63
S10	0.27	89	0.93	0.43	—

In order to confirm that PMKS can form dense crosslinked network phases in polysiloxanes, the average crosslinked densities of PMKS crosslinked polysiloxanes were studied and can be calculated according to eqn (7) for uniaxial extension.7
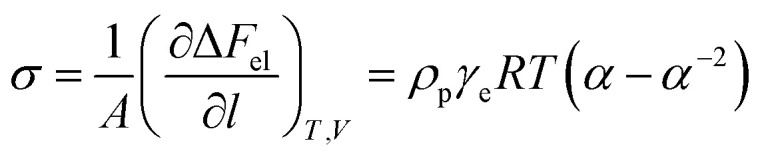
where *σ*, *A* represents the stress and the area of the sample, *α* stands for the elongation at break and *ρ*_p_ is density of cured specimen. The Helmholtz free energy of the network (Δ*F*_el_) can be expressed as follows^51^8
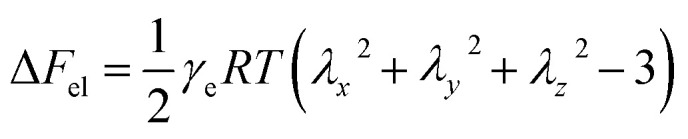
where *γ*_e_ is the crosslink density, *λ*_*i*_ (*i* = *x*, *y*, and *z*) represents the elongation in three dimensions, *T* is the ambient temperature, and *R* is gas constant.

**Fig. 9:**
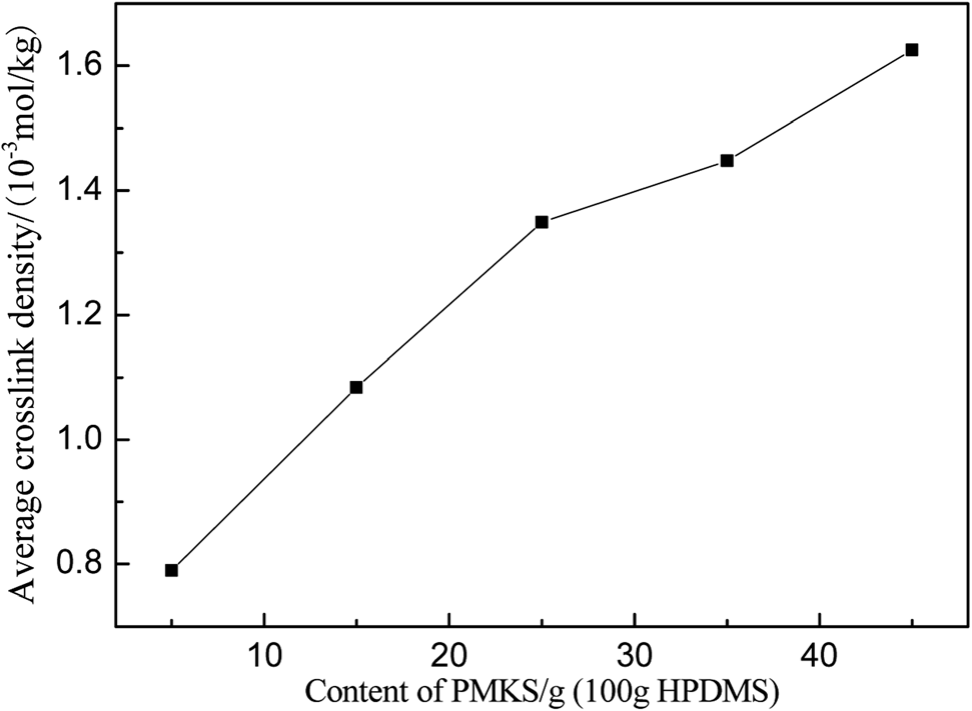
Average crosslink densities of PMKS/HPDMS as a function of PMKS content.

The average crosslink density can be calculated according to eqn (7) and depicted in Fig. 9 and Table 5. The average crosslink density increases as the loading of PMKS goes up, demonstrating that more dense PMKS phases are formed in the network. In the course of crosslinking reaction, excessive PMKS chains will form high crosslink density phased by the self-hydrolytic condensation of their rich pendant active groups. The hydroxyl-terminated PDMS chains turns to long chain networks because of the hydrolytic condensation between the hydroxyl group of HPDMS and the acetoxime groups of PMKS. As the molecular weight of PMKS (10^3^ g mol^−1^) is much lower than that of HPDMS (10^4^ g mol^−1^) and both of them are linear oligomers, HPDMS chain will form a loose crosslinking network with PMKS, whereas the residual PMKS turn to a dense phase by self-condensation and disperse into the continuous phase of HPDMS/PMKS. Therefore, PMKS dense phase might hinder the formation of cyclic oligomers and depress the oxidation degradation of carbon portion in polysiloxanes.

## 4. Conclusions

A novel cross-linker (PMKS) based on polymethylhydrosiloxane (PMHS), which was first synthesized successfully and applied to PDMS polymer system by chemical bonding with HPDMS polymer. The novel formulations of RTV silicone rubbers were prepared through hydrolysis-condensation under moisture between HPDMS and PMKS at 25 °C in the presence of catalyst. The thermal degradation and mechanical properties of the novel RTV silicone rubbers have been also studied. The TGA results showed that thermal decomposition of RTV silicone rubber containing PMKS took place in two steps, and the residual mass was higher than that of traditional crosslinking agent (MTKS). Furthermore, it is detected that dense PMKS phases blocked the formation of cyclic oligomers and reduced the oxidation degradation of carbon portion in polysiloxane during the decomposition in air. Besides, it was found that the novel PMKS cross-linker had a significant enhancement on thermal stability and mechanical properties as compared with traditional cross-linkers (MTKS). The observed improvement in thermal stability and mechanical properties could be attributed to the synergistic effect of the T-type branched structure of PMKS cross-linker and plasticization of dense PMKS phase *in situ* formed in the chain network.

## Conflicts of interest

There are no conflicts to declare.

## Supplementary Material
